# One-carbon metabolism is required for epigenetic stability in the mouse placenta

**DOI:** 10.3389/fcell.2023.1209928

**Published:** 2023-06-27

**Authors:** Claire E. Senner, Ziqi Dong, Malwina Prater, Miguel R. Branco, Erica D. Watson

**Affiliations:** ^1^ Centre for Trophoblast Research, University of Cambridge, Cambridge, United Kingdom; ^2^ Department of Physiology, Development, and Neuroscience, University of Cambridge, Cambridge, United Kingdom; ^3^ Department of Genetics, University of Cambridge, Cambridge, United Kingdom; ^4^ Centre for Genomics and Child Health, Blizard Institute, Faculty of Medicine and Dentistry, Queen Mary University of London, London, United Kingdom

**Keywords:** DNA methylation, folate, histone methylation, MTRR, sperm, transposable elements, trophoblast, epigenetic inheritance

## Abstract

One-carbon metabolism, including the folate cycle, has a crucial role in fetal development though its molecular function is complex and unclear. The hypomorphic *Mtrr* ^*gt*^ allele is known to disrupt one-carbon metabolism, and thus methyl group availability, leading to several developmental phenotypes (e.g., neural tube closure defects, fetal growth anomalies). Remarkably, previous studies showed that some of the phenotypes were transgenerationally inherited. Here, we explored the genome-wide epigenetic impact of one-carbon metabolism in placentas associated with fetal growth phenotypes and determined whether specific DNA methylation changes were inherited. Firstly, methylome analysis of *Mtrr* ^*gt/gt*^ homozygous placentas revealed genome-wide epigenetic instability. Several differentially methylated regions (DMRs) were identified including at the *Cxcl1* gene promoter and at the *En2* gene locus, which may have phenotypic implications. Importantly, we discovered hypomethylation and ectopic expression of a subset of ERV elements throughout the genome of *Mtrr* ^*gt/gt*^ placentas with broad implications for genomic stability. Next, we determined that known spermatozoan DMRs in *Mtrr* ^*gt*/*gt*^ males were reprogrammed in the placenta with little evidence of direct or transgenerational germline DMR inheritance. However, some spermatozoan DMRs were associated with placental gene misexpression despite normalisation of DNA methylation, suggesting the inheritance of an alternative epigenetic mechanism. Integration of published wildtype histone ChIP-seq datasets with *Mtrr* ^*gt/gt*^ spermatozoan methylome and placental transcriptome datasets point towards H3K4me3 deposition at key loci. These data suggest that histone modifications might play a role in epigenetic inheritance in this context. Overall, this study sheds light on the mechanistic complexities of one-carbon metabolism in development and epigenetic inheritance.

## 1 Introduction

It is well established that the vitamin folate (also known as folic acid) is important for fetal development. A highly recognisable example is increased risk of neural tube closure defects (e.g., spina bifida) in babies that result from maternal dietary folate deficiency (Emery et al., 1969). In fact, folic acid supplementation during pregnancy and folate fortification programmes improves pregnancy outcomes (MRC Vitamin Study Research Group, 1991; Gelineau-van Waes et al., 2008). Beyond the neural tube, other developmental defects [e.g., fetal growth restriction (Furness et al., 2008; Chen et al., 2018), congenital heart defects (Christensen et al., 2015)] and pregnancy disorders (Mislanova et al., 2011; Wu et al., 2015) are associated with dietary deficiency and/or mutations in key enzymes involved in its metabolism. Although well studied, the molecular role of folate metabolism during development is complex and not well understood. One-carbon metabolism, which includes the folate and methionine cycles, is required by all cells for thymidine synthesis and for methyl groups involved in a broad range of methylation reactions (Lin et al., 2022). As a result, it is hypothesised that rapidly proliferating cells in a developing fetus and placenta requires one-carbon metabolism for DNA synthesis and general epigenetic regulation. The specific genomic targets of one-carbon metabolism that drive developmental phenotypes remain unclear.

To explore the specific molecular role of one-carbon metabolism during development, we study a mouse model with a hypomorphic mutation in the methionine synthase reductase gene (*Mtrr* ^*gt*^) (Padmanabhan et al., 2013). During one-carbon metabolism, folate metabolites are required to transmit methyl groups for the methylation of homocysteine by methionine synthase (MTR) to form methionine and tetrahydrofolate (Shane and Stokstad, 1985). Methionine acts as precursor for S-adenosylmethionine (SAM), which in turn serves as the sole methyl-donor for substrates involved in epigenetic regulation (e.g., DNA, histones, RNA) among other substrates (Ducker and Rabinowitz, 2017). Importantly, MTRR activates MTR through the reductive methylation of its vitamin B_12_ co-factor (Leclerc et al., 1998; Yamada et al., 2006; Elmore et al., 2007). The hypomorphic *Mtrr* ^*gt*^ mutation reduces *Mtrr* transcript expression to a level that is sufficient to diminish MTR activity by 60% of controls (Elmore et al., 2007; Padmanabhan et al., 2013). Consequently, the progression of one-carbon metabolism is disrupted by the *Mtrr* ^*gt*^ mutation as evidenced by plasma hyperhomocysteinemia (Elmore et al., 2007; Padmanabhan et al., 2013) and widespread changes in DNA methylation patterns (Padmanabhan et al., 2013; Bertozzi et al., 2021; Blake et al., 2021). Additionally, *Mtrr* ^*gt/gt*^ mice display several phenotypes similar to the clinical features of folate deficiency in humans (Krishnaswamy and Madhavan Nair, 2001) or human *MTRR* mutations (Schuh et al., 1984; Wilson et al., 1999) including macrocytic anemia (Padmanabhan et al., 2018) and neural tube closure defects (NTDs) (Padmanabhan et al., 2013; Wilkinson et al., 2021). Beyond this, other phenotypes have emerged in *Mtrr* ^*gt/gt*^ mice reflecting a broader influence of impaired one-carbon metabolism on development. These phenotypes include fetal growth defects (such as fetal growth restriction (FGR), fetal growth enhancement (FGE), or developmental delay) (Padmanabhan et al., 2013; Padmanabhan et al., 2017), complications during implantation (e.g., twinning, skewed implantation) (Padmanabhan et al., 2013; Wilkinson et al., 2021), haemorrhages, and/or congenital malformations (such as congenital heart defects and poor placentation) (Deng et al., 2008; Padmanabhan et al., 2013; Wilkinson et al., 2021). Therefore, the *Mtrr* ^*gt*^ mouse line is ideal for exploring the molecular consequences of defective one-carbon metabolism during growth and development.

Remarkably, the *Mtrr* ^*gt*^ mouse line is also a unique mammalian model of transgenerational epigenetic inheritance that occurs via the maternal grandparental lineage (Padmanabhan et al., 2013; Blake et al., 2021). Through highly controlled genetic pedigrees and embryo transfer experiments, we previously showed that an *Mtrr* ^*+/gt*^ genotype in male or female mice (i.e., the F0 generation) initiates multigenerational inheritance of developmental phenotypes in their wildtype (*Mtrr* ^*+/+*^) grandprogeny (i.e., the F2–F4 generations) (Padmanabhan et al., 2013; Padmanabhan et al., 2017). This effect occurs through their F1 wildtype daughters (Padmanabhan et al., 2013). In general, the mechanism of epigenetic inheritance is not well understood. In the context of the *Mtrr* ^*gt*^ mouse line, we hypothesise that alterations in the epigenome of the F0 germline caused by abnormal one-carbon metabolism is inherited by the wildtype offspring of the next generation (and potentially beyond) to influence gene expression during development (Padmanabhan et al., 2013; Blake and Watson, 2016; Blake et al., 2021). Given the role of MTRR in one-carbon metabolism, and thus in cellular methylation, we initially focused on how the *Mtrr* ^*gt*^ mutation alters DNA methylation patterns across generations. Through a targeted analysis, we previously determined that developmental phenotypes at E10.5 in *Mtrr* ^*gt/gt*^ conceptuses or F2 *Mtrr* ^*+/+*^ conceptuses derived by an F0 *Mtrr* ^*+/gt*^ maternal grandparent were associated with locus-specific changes in DNA methylation linked to gene misexpression (Padmanabhan et al., 2013; Blake et al., 2021). The effect was particularly striking in the placenta at key genes involved in the regulation of fetal growth and metabolism (Padmanabhan et al., 2013). It was also clear that epigenetic instability of DNA methylation occurs in mature spermatozoa from *Mtrr* ^+/*gt*^ and *Mtrr* ^*gt*/*gt*^ males as well as from F1 *Mtrr* ^+/+^ male progeny of F0 *Mtrr* ^+/*gt*^ males (Blake et al., 2021), which otherwise display normal spermatogenesis and spermatozoa function (Blake et al., 2019). However, the extent to which these altered germline methylation patterns are recapitulated in (or inherited by) the somatic cells of the progeny and grandprogeny is currently not well understood in the *Mtrr* ^*gt*^ mouse line.

In this study, we use genome-wide approaches to investigate the global impact of one-carbon metabolism on the placental methylome in *Mtrr* ^*gt/gt*^ homozygous mice and in F2 *Mtrr* ^*+/+*^ mice derived from F0 *Mtrr* ^*+/gt*^ maternal grandfathers. In doing so, we probe the underlying impact on fetal growth and whether the differentially methylated regions (DMRs) are functionally important and/or inherited. We reveal that the *Mtrr* ^*gt/gt*^ placental methylome is unstable with implications for phenotype establishment, transposable element regulation, and genetic stability. We also determine that specific DMRs observed in spermatozoa of *Mtrr* ^*gt/gt*^ males are reprogrammed in *Mtrr* ^*gt/gt*^ placentas and as a result, we explore other epigenetic mechanisms for inheritance including histone methylation (H3K4me3). We integrate published ChIP-seq datasets from wildtype embryonic and trophoblast lineages with our *Mtrr* ^*gt/gt*^ spermatozoan methylome and placental transcriptome datasets. Overall, these analyses delve into the mechanistic complexities of one-carbon metabolism during development and epigenetic inheritance of phenotype.

## 2 Materials and methods

### 2.1 Ethics statement

This research was regulated under the Animals (Scientific Procedures) Act 1986 Amendment Regulations 2012 following ethical review by the University of Cambridge Animal Welfare and Ethical Review Body.

### 2.2 Mouse model

*Mtrr* ^*Gt(XG334)Byg*^ (MGI:3526159) mouse line, referred to as the *Mtrr* ^*gt*^ mouse line, was generated when a *β-geo* gene-trap (gt) vector was inserted into intron 9 of the *Mtrr* gene in 129P2Ola/Hsd embryonic stem cells (ESCs) (Elmore et al., 2007; Padmanabhan et al., 2013). *Mtrr* ^*gt*^ ECSs were injected into C57Bl/6J blastocysts and upon germline transmission, the *Mtrr* ^*gt*^ allele was backcrossed into the C57Bl/6J genetic background for at least eight generations (Padmanabhan et al., 2013). *Mtrr* ^*+/+*^ and *Mtrr* ^*+/gt*^ mice were generated from *Mtrr* ^*+/gt*^ intercrosses. *Mtrr* ^*gt/gt*^ mice were generated by *Mtrr* ^*gt/gt*^ intercrosses. Since the *Mtrr* ^*gt*^ allele has a multigenerational effect (Padmanabhan et al., 2013; Bertozzi et al., 2021; Blake et al., 2021), C57Bl/6J mice from The Jackson Laboratories (www.jaxmice.jax.org) were used as controls and were bred in-house and maintained separately from the *Mtrr* ^*gt*^ mouse line. The effects of the maternal grandpaternal *Mtrr* ^*gt*^ allele were determined by the following pedigree: F0 *Mtrr* ^*+/gt*^ males were mated to C57Bl/6J females. The resulting F1 *Mtrr* ^*+/+*^ females were mated to C57Bl/6J males to generate F2 *Mtrr* ^*+/+*^ conceptuses. Genotyping for *Mtrr* ^*+*^ and *Mtrr* ^*gt*^ alleles was performed using PCR on DNA extracted from ear tissue or yolk sac using a three-primer reaction resulting in a wildtype band at 252 bp and a mutant band at 383 bp (Padmanabhan et al., 2013). Primer sequences: primer *a* (5′-GAG​ATT​GGG​TCC​CTC​TTC​CAC), primer *b* (5′-GCT​GCG​CTT​CTG​AAT​CCA​CAG), and primer *c* (5′-CG ACT​TCC​GGA​GCG​GAT​CTC) (Padmanabhan et al., 2013). All mice were housed in a temperature-and humidity-controlled environment with a 12 h light-dark cycle. All mice were fed a normal chow diet (Rodent No. 3 chow, Special Diet Services) *ad libitum* from weaning, which included (per kg of diet): 1.6 g choline, 2.73 mg folic acid, 26.8 μg vitamin B_12_, 3.4 g methionine, 51.3 mg zinc.

### 2.3 Dissections and tissue collection

Noon of the day that the vaginal plug was detected was defined as embryonic (E) day 0.5. Mice were euthanized by cervical dislocation. Fetuses and placentas were dissected in cold 1x phosphate buffered saline (PBS) at E10.5 using a Zeiss SteReo Discovery V8 microscope, scored for phenotypes, and photographed. Fetuses and placentas were weighed and measured separately and snap frozen in liquid nitrogen (stored at −80°C). Both male and female placentas were assessed since no phenotypic sexual dimorphism was identified at E10.5 (Padmanabhan et al., 2017).

### 2.4 Phenotyping

Conceptuses were rigorously scored for gross phenotypes during dissection and allocated to the phenotypic categories that were previously defined, including phenotypically normal (PN), fetal growth enhancement (FGE), fetal growth restriction (FGR), developmental delay, severe abnormalities (e.g., congenital heart defects, neural tube closure defects, hemorrhages, skewed conceptus orientation, twinning, etc.), and resorption (Padmanabhan et al., 2013; Wilkinson et al., 2021). Notably, conceptuses with >1 phenotype were counted once and classified by the most severe phenotype observed. Only PN, FGR, and FGE conceptuses were assessed in this study. Phenotype parameters are defined below.

#### 2.4.1 PN conceptuses

Fetuses and placentas met all developmental milestones appropriate for the developmental stage according to e-Mouse Atlas Project (https://www.emouseatlas.org/emap/home.html). PN fetuses at E10.5 contained 30–39 somite pairs and had crown-rump lengths that were within two standard deviations (sd) from the mean of C57Bl/6J fetuses at E10.5, putting them within the normal range for growth. All PN conceptuses lacked abnormalities identified via gross assessment.

#### 2.4.2 FGR and FGE conceptuses

Conceptuses with FGR and FGE lacked abnormalities identified via gross dissection and met the staging criteria for E10.5 (i.e., 30–39 somite pairs). Yet, the fetuses displayed crown-rump lengths that were ≥2 sd below (for FGR) or above (for FGE) the mean crown-rump length for C57Bl/6J fetuses (Padmanabhan et al., 2013). Conceptus size was unaffected by litter size in all pedigrees and stages assessed (Padmanabhan et al., 2017).

### 2.5 Methylated DNA immunoprecipitation (meDIP) and next-generation sequencing

Whole placentas at E10.5 were homogenized using a MagNA Lyser Instrument (Roche) and incubated on an Eppendorf ThermoMixer at 1,000 rpm at 56°C for 10 min. Genomic DNA was extracted using a QIAamp Fast DNA Tissue kit (Qiagen) following the manufacturer’s instructions. MeDIP-Seq was carried out as described previously (Ficz et al., 2011). Briefly, genomic DNA was sonicated to yield 150–600 bp fragments, and adaptors for paired-end sequencing (Illumina) were ligated using the NEBNext Ultra II DNA Library Prep Kit for Illumina (New England Biolabs). Immunoprecipitations were carried out using 500 ng DNA per sample, 1.25 μg anti-5mC antibody (Eurogentec Cat# BI-MECY-0100, RRID:AB_2616058) or mouse immunoglobulin G (IgG) control and 10 μL Dynabeads coupled with M-280 sheep anti-mouse antibody (Invitrogen). Pulled down DNA was amplified for 12 cycles (meDIP) or 15 cycles (IgG control) with adapter-specific indexed primers. Final clean-up and size selection was carried out with AMPure-XP SPRI beads (Beckman Coulter). Libraries were quantified and assessed using the Kapa Library Quantification Kit (Kapa Biosystems) and Bioanalyzer 2100 System (Agilent). Indexed libraries were sequenced (50-bp paired-end) on an Illumina HiSeq 2500 sequencer. Raw fastq data were trimmed with TrimGalore (v0.6.6), using default parameters, and unique reads mapped to the *Mus musculus* GRCm38 genome assembly using Bowtie2 (v2.4.1). Data analysis was carried out using SeqMonk software (www.bioinformatics.babraham.ac.uk).

### 2.6 RNA-sequencing

Whole male placentas at E10.5 were homogenized using lysing matrix D beads. RNA library preparation and sequencing was performed by Cambridge Genomic Services, Department of Pathology, University of Cambridge. The concentration and purity of RNA was determined by a SpectroStar spectrophotometer (BMG LABTECH) and the RNA integrity was determined by an Agilent Tapestation Bioanalyzer (Aligent Technologies LDA United Kingdom Ltd.). Libraries were prepared using 200 ng of total RNA and TruSeq stranded mRNA Library Preparation kit (Illumina). A unique index sequence was added to each RNA library to allow for multiplex sequencing. Libraries were pooled and sequenced on the Illumina NextSeq500 platform with 75 bp single-end reads. Sequencing was performed in duplicate to provide >18 million reads per sample. To monitor sequencing quality control, 1% PhiX Control (Illumina) spike-in was used. Quality control of Fastq files was performed using FastQC and fastq_screen. Sequences were trimmed with Trim Galore! and aligned to GRCm38 mouse genome using STAR aligner. Alignments were processed using custom ClusterFlow (v0.5dev) pipelines and assessed using MultiQC (0.9. dev0). Gene quantification was determined with HTSeq- Counts (v0.6.1p1). Additional quality control was performed with rRNA and mtRNA counts script, feature counts (v 1.5.0- p2) and qualimap (v2.2). Differential gene expression was performed with DESeq2 package (v1.22.2, R v3.5.2). Read counts were normalised on the estimated size factors.

### 2.7 Transposable element analysis

To include transposon-derived reads that do not map uniquely, the meDIP-seq datasets were re-aligned using the default settings of bowtie2 to assign reads with multiple equally best alignments to one of those locations at random. Average methylation levels over pro-viral, full-length elements were generated after merging Repeatmasker annotations for *RLTR4_Mm* and *RLTR4_MM-int* elements. RNA-seq data was analysed using SQuIRE (Yang et al., 2019), which assigns multimapping reads using an expectation-maximisation algorithm and provides both subfamily-level and single copy-level information. Differential expression analysis was performed using SQuIRE’s Call function.

## 3 Results

### 3.1 Global analysis of the *Mtrr* ^*gt/gt*^ placenta methylome

**FIGURE 1:**
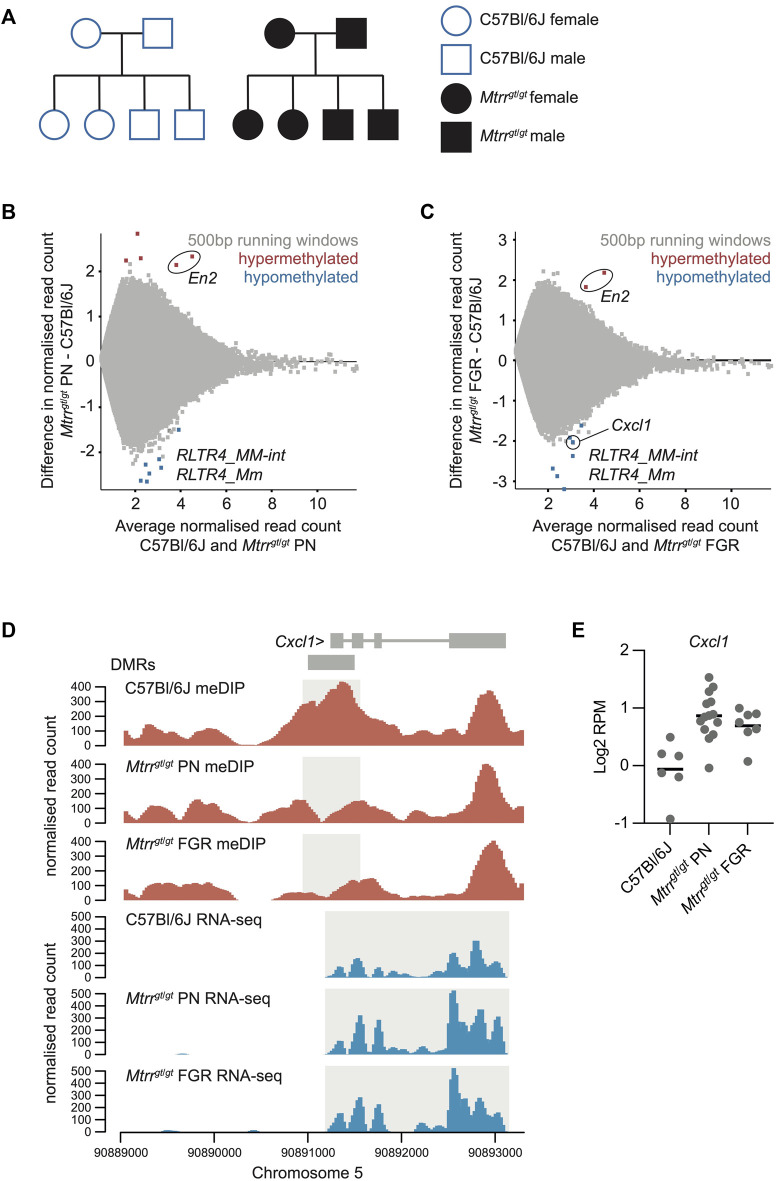
Analysis of the *Mtrr* ^*gt/gt*^ placental methylome. **(A)** Schematization of the C57Bl6J control pedigree (blue outline, white fill) and *Mtrr* ^*gt*/*gt*^ pedigree (black outline, black fill) used in this study. Square, males; Circles, females. **(B,C)** MA plot of *log*_*2*_ normalized meDIP-seq read counts of 500 bp contiguous regions in **(B)** C57Bl/6J placentas and *Mtrr* ^*gt*/*gt*^ placentas from phenotypically normal (PN) fetuses, and **(C)** C57Bl/6J placentas and *Mtrr* ^*gt*/*gt*^ placentas associated with fetal growth restriction (FGR). Hypermethylated (red) and hypomethylated (blue) differentially methylated 500 bp regions (DMR) were identified using EdgeR. **(D)** Data tracks showing normalized meDIP-seq (red) and RNA-seq (blue) reads across the *Cxcl1* locus on mouse chromosome 5 in C57Bl/6J, *Mtrr* ^*gt*/*gt*^ PN and *Mtrr* ^*gt*/*gt*^ FGR placentas. DMR and transcript expression are highlighted in light grey. **(E)** Graph showing *Cxcl1* transcript expression (*log*_*2*_RPM) ascertained by RNA-seq in C57Bl/6J, *Mtrr* ^*gt*/*gt*^ PN, and *Mtrr* ^*gt*/*gt*^ FGR placentas at E10.5. In all cases data was normalized to the largest data store. For meDIP-seq: C57Bl/6J, *N* = 8 placentas; *Mtrr* ^*gt/gt*^ PN, *N* = 7 placentas; *Mtrr* ^*gt/gt*^ FGR, *N* = 7 placentas. For RNA-seq: C57Bl/6J, *N* = 6 placentas; *Mtrr* ^*gt/gt*^ PN, *N* = 14 placentas; *Mtrr* ^*gt/gt*^ FGR, *N* = 7 placentas.

First, we analysed the extent to which impaired one-carbon metabolism affected the placental methylome and ascertained whether there was an impact on fetal growth. Our initial focus was on *Mtrr* ^*gt*/*gt*^ placentas of conceptuses derived from *Mtrr* ^*gt/gt*^ intercrosses (Figure 1A). We carried out high-throughput sequencing of immunoprecipitated methylated DNA (meDIP-seq) from C57Bl/6J control and *Mtrr* ^*gt*/*gt*^ placentas at E10.5. *Mtrr* ^*gt/gt*^ placentas were divided into two phenotypic groups including those from fetuses that were phenotypically normal (PN) or were FGR based on crown-rump length (Padmanabhan et al., 2013; Wilkinson et al., 2021). Placentas from C57Bl/6J mice were controls since the *Mtrr* ^*gt*^ allele was backcrossed into the C57Bl/6J genetic background (Padmanabhan et al., 2013). However, we previously identified four regions of structural variation between C57Bl/6J and the *Mtrr* ^*gt*^ line (Blake et al., 2021). To avoid false discovery of changes in DNA methylation during the meDIP-seq data analysis, these regions were excluded bioinformatically along with the 20 Mb region of 129P2Ola/Hsd genomic sequence surrounding the gene-trapped *Mtrr* allele (Bertozzi et al., 2021; Blake et al., 2021), that remained after eight backcrosses (Padmanabhan et al., 2013).

At the global level, we found that the distribution of meDIP-seq reads across different genomic features were not significantly different between C57Bl/6J control and *Mtrr* ^*gt*/*gt*^ placentas even when phenotypic severity was considered (Supplementary Figure S1A). Furthermore, meDIP-seq datasets from individual placentas did not cluster by *Mtrr* genotype or fetal growth phenotype when data store similarity tools were implemented (Supplementary Figure S1B). As global DNA methylation patterns were similar between experimental groups, we next ascertained differences in DNA methylation at individual loci compared to control placentas. DMRs were defined using the EdgeR function embedded within Seqmonk software (www.bioinformatics.babraham.ac.uk) with default settings (*p* < 0.05, with multiple testing correction) assessing 500 bp contiguous regions. The resulting DMRs were further filtered for regions that displayed a *log*_*2*_ fold change (FC) > 1 in DNA methylation compared to controls. Only a few DMRs were present in *Mtrr* ^*gt*/*gt*^ placentas (i.e., PN: 13 DMRs; FGR: 9 DMRs), though both hyper- and hypomethylated regions were observed (Figures 1B, C; Supplementary File S1). The low number of DMRs caused by the *Mtrr* ^*gt*^ allele suggested that DNA methylation changes were subtle regardless of fetal growth phenotypes or were “hidden” by our analysis of whole placentas as individual cell types might be differently affected.

Despite the low number of total DMRs, three key findings emerged (explored further below). Firstly, only one placental DMR associated with the misexpression of a protein-coding gene (*Cxcl1*; Figures 1D, E). Secondly, we identified two hypermethylated DMRs located within the *En2* gene in *Mtrr* ^*gt*/*gt*^ placentas that were common to PN and FGR conceptuses (Figures 1B, C). Since the *En2* DMRs were also identified in mature spermatozoa from *Mtrr* ^*gt/gt*^ males and in *Mtrr* ^*gt/gt*^ embryos at E10.5 (Blake et al., 2021), they were flagged for further analysis in the context of development and epigenetic inheritance. Lastly, of the 12 hypomethylated DMRs that were identified (i.e., 3 shared DMRs in PN and FGR placentas, 5 DMRs in PN placentas only, 4 DMRs in FGR placentas only), ten were associated with endogenous retroviruses (ERVs; Supplementary File S1). Strikingly, the majority of these DMRs (7/10) overlapped with ERV1 elements of the RLTR4 subclass (Figures 1B, C) with implications for genetic stability. Further exploration into the importance of these findings was explored below.

### 3.2 Potential canonical regulation of placental *Cxcl1* expression by DNA methylation

To explore whether altered placental DNA methylation caused by the *Mtrr* ^*gt/gt*^ genotype had a gene regulatory effect, we carried out RNA-seq on *Mtrr* ^*gt/gt*^ placentas at E10.5 associated with PN and FGR fetuses. Using DESeq, the RNA-seq data was assessed for differentially expressed genes that were within 2 kb of a DMR (identified in *Mtrr* ^*gt/gt*^ placentas) and had transcript levels with a *log*_*2*_FC > 0.6 compared to control placentas. The *Cxcl1* gene [chemokine (C-X-C motif) ligand 1] was the only dysregulated gene identified in this context. We observed that hypomethylation at the DMR located in the promoter of *Cxcl1* was associated with a modest upregulation of *Cxcl1* transcripts (Figures 1D, E). This finding exemplifies canonical regulation of a gene by DNA methylation. Since *Mtrr* ^*gt/gt*^ placentas from both PN and FGR fetuses displayed hypomethylation at the *Cxcl1* DMR and upregulation of *Cxcl1* transcripts (Figures 1D, E), these molecular changes were likely insufficient to drive the fetal growth phenotype. Yet, CXCL1 is important for decidual angiogenesis to promote maternal blood flow into the implantation site (Ma et al., 2021). Therefore, dysregulation of *Cxcl1* mRNA in *Mtrr* ^*gt/gt*^ placentas might have implications for fetoplacental development beyond fetal growth.

### 3.3 *En2* DMR as a potential regulator of developmentally important genes

**FIGURE 2:**
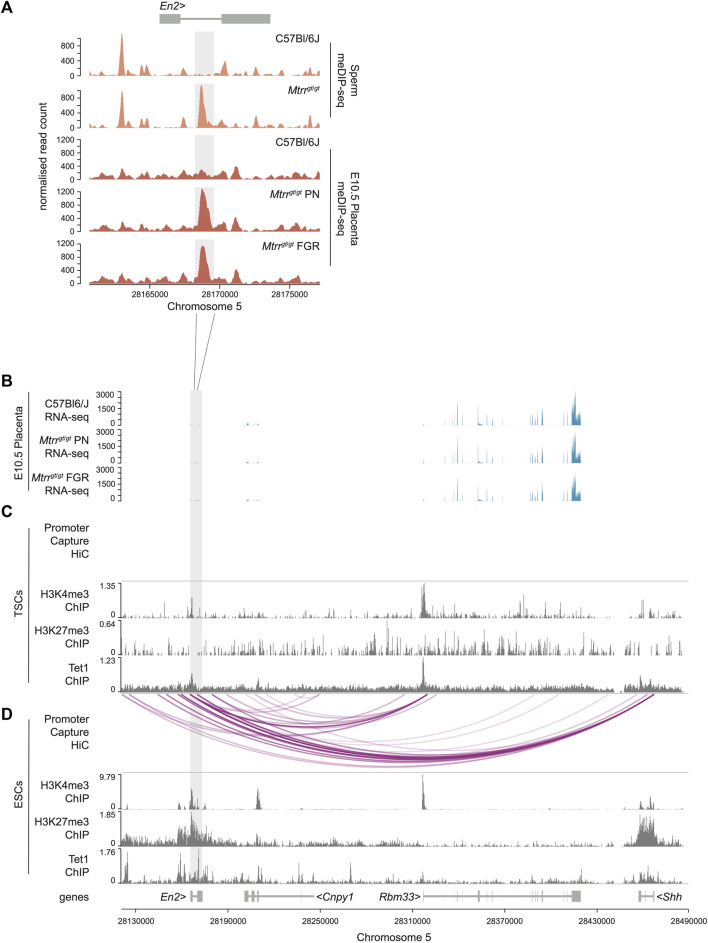
*En2* DMR as a potential regulator of developmentally important genes in ESCs and not TSCs. **(A)** Data tracks showing normalized meDIP-seq read counts across the *En2* gene in spermatozoa from C57BL/6J and *Mtrr* ^*gt*/*gt*^ mice (orange) and placentas from C57Bl/6J and *Mtrr* ^*gt*/*gt*^ conceptuses at E10.5 (red). Placentas were associated with either phenotypically normal (PN) or fetal growth restricted (FGR) fetuses. The *En2* DMR is highlighted in light grey. **(B)** RNA-seq data tracks showing gene expression (blue) in the genomic region near to the *En2* DMR (light grey) in placentas at E10.5 from C57Bl/6J and *Mtrr* ^*gt*/*gt*^ conceptuses. Placentas from PN and FGR fetuses were considered. **(C,D)** Data tracks showing complete promoter capture HiC-based interactions (purple lines) and H3K27me3, H3K4me3 and Tet1 ChIP-seq peaks (dark grey) at the *En2* locus and downstream genes within **(C)** wildtype mouse trophoblast stem cells (TSCs) and **(D)** wildtype mouse embryonic stem cells (ESCs). See also Supplementary File S3 for data sources. For sperm meDIP-seq: C57Bl/6J, *N* = 8 males; *Mtrr* ^*gt/gt*^, *N* = 8 males. For placenta meDIP-seq: C57Bl/6J, *N* = 8 placentas; *Mtrr* ^*gt/gt*^ PN, *N* = 7 placentas; *Mtrr* ^*gt/gt*^ FGR, N = 7 placentas.

The only two hypermethylated DMRs identified in *Mtrr* ^*gt/gt*^ placentas were found within the *En2* gene. Therefore, their functional importance was explored. These two 500 bp DMRs were in fact contiguous and represented one single 1 kb region in the single *En2* intron (Figure 2A). The *En2* gene encodes a homeobox transcription factor that, when knocked out in mice, leads to autism-spectrum disease-like behaviours (Cheh et al., 2006; Brielmaier et al., 2012; Provenzano et al., 2014) that are accompanied by cerebellar foliation defects (Joyner et al., 1991) and loss of GABAergic interneurons in somatosensory and visual cortical areas (Sgado et al., 2013; Allegra et al., 2014). Indeed, *En2* mRNA is expressed in multiple regions of the developing brain (Davis et al., 1988) and is involved in neurogenesis (Lee et al., 1997). Low levels of *En2* transcripts were reported by RNA-seq in the ectoplacental cone (Bastian et al., 2021) (a population of trophoblast progenitor cells in the mouse placenta). However, our RNA-seq data from whole C57Bl/6J control placentas at E10.5 showed that *En2* transcripts were very lowly expressed (Figure 2B) and thus, *En2* might be considered as an unexpressed gene in the placenta at this developmental stage. Importantly, hypermethylation of the *En2* DMR in *Mtrr* ^*gt/gt*^ placentas was not associated with a change in *En2* transcript levels (Figure 2B) indicating that the *En2* DMR is an unlikely regulator of *En2* gene expression in the placenta.

To investigate a broader regulatory role of the *En2* DMR in the placenta, we explored histone methylation (e.g., H3K4me3 and H3K27me3) enrichment and potential interactions of the DMR with neighbouring genes. To do this, we analysed published H3K4me3 and H3K27me3 ChIP-seq datasets and promoter capture Hi-C datasets from wildtype mouse trophoblast stem cells (TSCs) (Schoenfelder et al., 2018). TSCs are an *in vitro* model of undifferentiated trophoblast cells of the placenta (Tanaka et al., 1998), and these datasets represent the most suitable available for analysis. No enrichment of H3K4me3 or H3K27me3 modifications was evident at the *En2* DMR in TSCs and no complete DMR-promoter interactions were evident in TSCs within the genomic region assessed (Figures 2B, C). Accordingly, genes downstream of the *En2* DMR were also expressed at normal levels in *Mtrr* ^*gt/gt*^ placentas (Figure 2B) indicating that hypermethylation of this region had little to no effect on cis regulation of gene expression in the placenta.

The hypermethylated *En2* DMR is prevalent in different tissue types including mature spermatozoa of *Mtrr* ^*gt/gt*^ males and *Mtrr* ^*gt/gt*^ embryos and placentas at E10.5 (Blake et al., 2021; this study). Given that the *En2* gene is important for the development of embryonic lineages [e.g., neurogenesis (Lee et al., 1997)], the potential regulatory importance of the *En2* DMR was explored outside of the placenta. Additional ChIP-seq and promoter capture Hi-C datasets from wildtype mouse ESCs (Schoenfelder et al., 2018) were analysed in the region proximal to the *En2* DMR. The data revealed that the *En2* DMR had hallmarks of a regulatory locus in ESCs since it was bivalently marked by the enrichment of repressive H3K27me3 and active H3K4me3 modifications (Figure 2D) in a manner that poises this region for activation upon cell differentiation (Macrae et al., 2023). The genomic region defined by the *En2* DMR in ESCs was also enriched for the DNA demethylating enzyme TET1 (Figure 2D), which typically co-localises with polycomb complexes and contributes to keeping unmethylated enhancers and promoters methylation-free (Parry et al., 2021). Furthermore, promoter capture Hi-C experiments in ESCs (Schoenfelder et al., 2018) revealed a potential interaction of the *En2* DMR with the promoters of nearby genes, including *Cnpy1* (canopy FGF signalling regulator 1), *Rbm33* (RNA binding motif 33), and the developmental regulator *Shh* (sonic hedgehog) (Figure 2D). These data contrasted the TSCs data, which showed no such DMR-promoter interactions (Figure 2C). Therefore, we hypothesised that ectopic hypermethylation of the En2 DMR specifically within *Mtrr* ^*gt/gt*^ embryos might affect expression of surrounding genes with developmental consequences. Further analysis of the developmental role of the *En2* DMR in the embryo is required, particularly in the context of abnormal one-carbon metabolism.

### 3.4 Hypomethylation and ectopic expression of ERVs indicates epigenetic instability in the *Mtrr* ^*gt*^ mouse line

**FIGURE 3:**
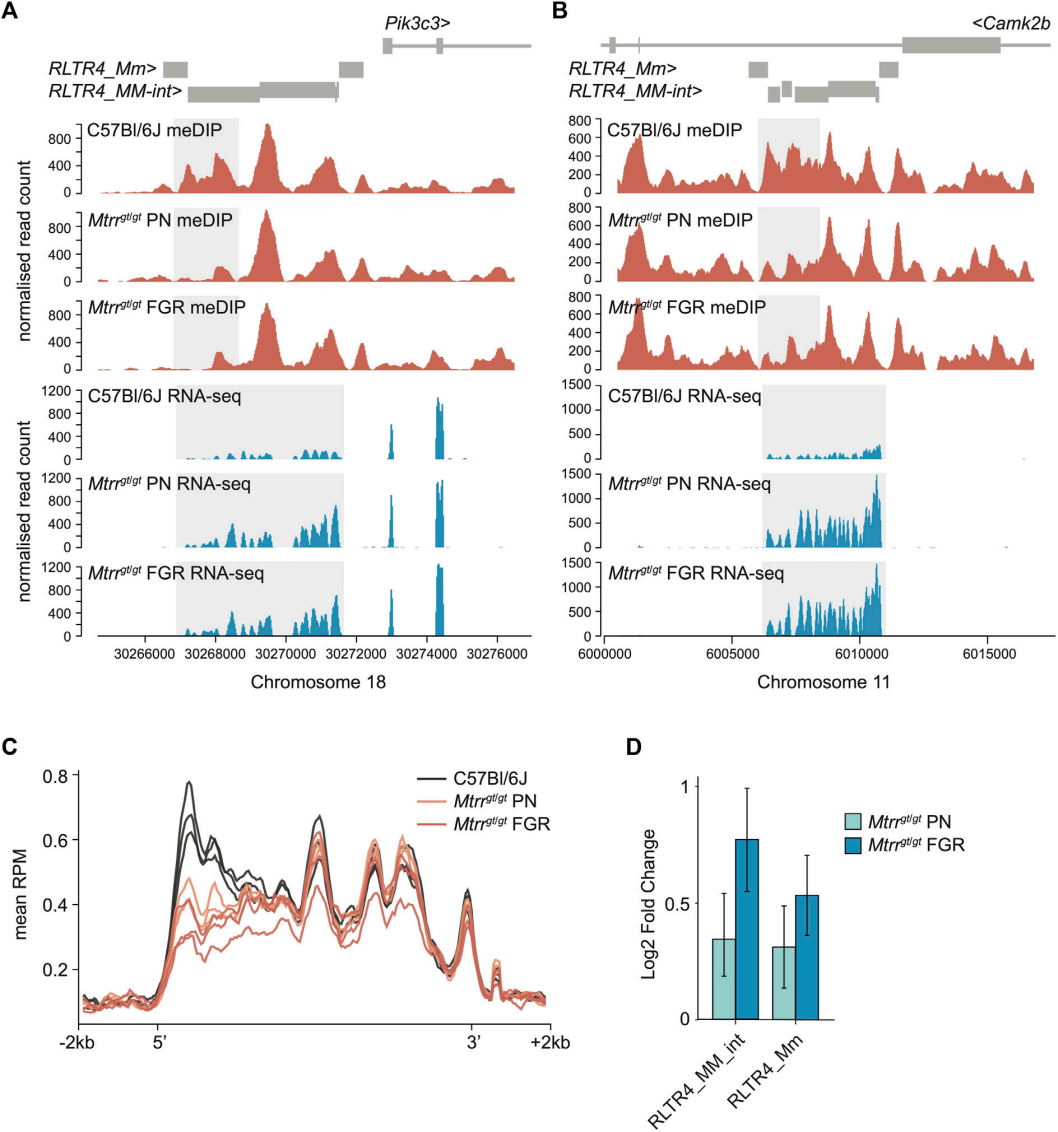
Analysis of DNA methylation and transcript expression at ERV subfamily in *Mtrr* ^*gt/gt*^ placentas. **(A,B)** Data tracks showing normalized meDIP-seq (red) and RNA-seq (blue) reads across full-length ERVs comprising *RLTR4_Mm* and *RLTR4_MM-int* elements on mouse **(A)** chromosome 18 associated with the *Pik3c3* gene and **(B)** chromosome 11 associated with the *Camk2b* gene in placentas of C57Bl/6J and *Mtrr* ^*gt*/*gt*^ conceptuses at E10.5. Placentas from phenotypically normal (PN) and fetal growth restricted (FGR) fetuses were assessed. Differentially methylated region (DMR) and transcript expression are highlighted in light grey. **(C)** Graph representing the average meDIP-seq reads mapping across all full-length RLTR4 elements ± 2 kb in the genome in individual placentas from C57Bl/6J (black), *Mtrr* ^*gt/gt*^ PN (orange) and *Mtrr* ^*gt/gt*^ FGR (red) fetuses at E10.5. **(D)** Enrichment of *RLTR4_Mm* and *RLTR4_MM-int* expression in placentas from *Mtrr* ^*gt*/*gt*^ PN (light blue) and *Mtrr* ^*gt*/*gt*^ FGR (dark blue) fetuses at E10.5 relative to C57Bl/6J control placentas as determined by RNA-seq. For meDIP-seq: C57Bl/6J, *N* = 8 placentas; *Mtrr* ^*gt/gt*^ PN, *N* = 7 placentas; *Mtrr* ^*gt/gt*^ FGR, *N* = 7 placentas. For RNA-seq: C57Bl/6J, *N* = 6 placentas; *Mtrr* ^*gt/gt*^ PN, *N* = 14 placentas; *Mtrr* ^*gt/gt*^ FGR, *N* = 7 placentas.

We identified ten hypomethylated DMRs in *Mtrr* ^*gt*/*gt*^ placentas at E10.5 that were associated with ERV elements (Supplementary File S1). Specifically, seven of these overlapped with RLTR4 elements of the ERV1 subfamily (separately annotated as *RLTR4_Mm* and *RLTR4_MM-int* for the LTRs and internal region, respectively; Figures 1B, C). RLTR4 elements are relatively young retrotransposons that are closely related to murine leukemia virus and that, at least in some mouse strains, remain transpositionally active (Maksakova et al., 2006). Some transposable elements (e.g., IAPs) are highly methylated and resistant to epigenetic reprogramming to avoid genomic transposition (Kobayashi et al., 2012). It is unclear whether this is the case for RLTR4 elements. The RLTR4 elements that associated with placental DMRs in this study typically displayed a pro-viral, full-length configuration, rather than being solo LTRs or other isolated fragments. Furthermore, six out of seven of the RLTR4-associated DMRs mapped to two discrete genomic loci on mouse chromosomes 11 or 18, including in regions that were intragenic (and antisense) to *Camk2b* or were upstream of the gene *Pik3c3*, respectively (Figures 3A, B). Remarkably, a loss of DNA methylation at these DMRs corresponded with ectopic expression of the RLTR4 element in *Mtrr* ^*gt/gt*^ placentas, independent of the fetal growth phenotype (Figures 3A, B). However, no expression changes in the associated protein-coding genes were observed (Figures 3A, B). Therefore, the genomic regions demarcated by these DMRs appear to require methylation to repress the ERV element activity and not to regulate cis gene expression in the placenta.

Due to their repetitive nature and evolutionary young age, the mapability of short sequencing reads to RLTR4 elements is low. Therefore, to fully appreciated the dysregulation of DNA methylation at RLTR4 elements in *Mtrr* ^*gt/gt*^ placentas, the placental meDIP-seq and RNA-seq datasets from C57Bl/6J and *Mtrr* ^*gt/gt*^ placentas at E10.5 were re-mapped to include non-unique reads by using random assignment (bowtie2) for meDIP-seq data and an expectation-maximisation algorithm [SQuIRE (Yang et al., 2019)] for RNA-seq data. When considered globally, the remapped data revealed consistent DNA hypomethylation at the 5′ end of RLTR4 full-length elements in *Mtrr* ^*gt*/*gt*^ placentas at E10.5 (Figure 3C). This pattern of DNA hypomethylation was associated with transcript enrichment of global *RLTR4_Mm* and *RLTR4_MM-int* elements in *Mtrr* ^*gt*/*gt*^ placentas compared to controls (Figure 3D). Differential expression analysis of individual elements uncovered significant upregulation of twenty-four *RLTR4_Mm* or *RLTR4_MM-int* elements that converged upon 15 full-length loci (Supplementary File S2). None of these methylation changes associated with altered expression of nearby protein-coding genes in *Mtrr* ^*gt/gt*^ placentas. Furthermore, methylation and transcriptional dysregulation at *RLTR4_Mm* and *RLTR4_Mm-int* elements was unlikely to regulate fetal growth since the RLTR4 elements were similarly affected in *Mtrr* ^*gt/gt*^ placentas associated with PN and FGR fetuses (Figure 3D). Overall, these data reinforced the hypothesis that epigenetic instability is inherent to the *Mtrr* ^*gt*^ mouse line (Padmanabhan et al., 2013; Blake et al., 2021) with implications for genetic stability and phenotype establishment beyond FGR.

### 3.5 Mature germ cell DMRs in *Mtrr* ^*gt/gt*^ males are reprogrammed in the placenta but correspond to gene misexpression

**FIGURE 4:**
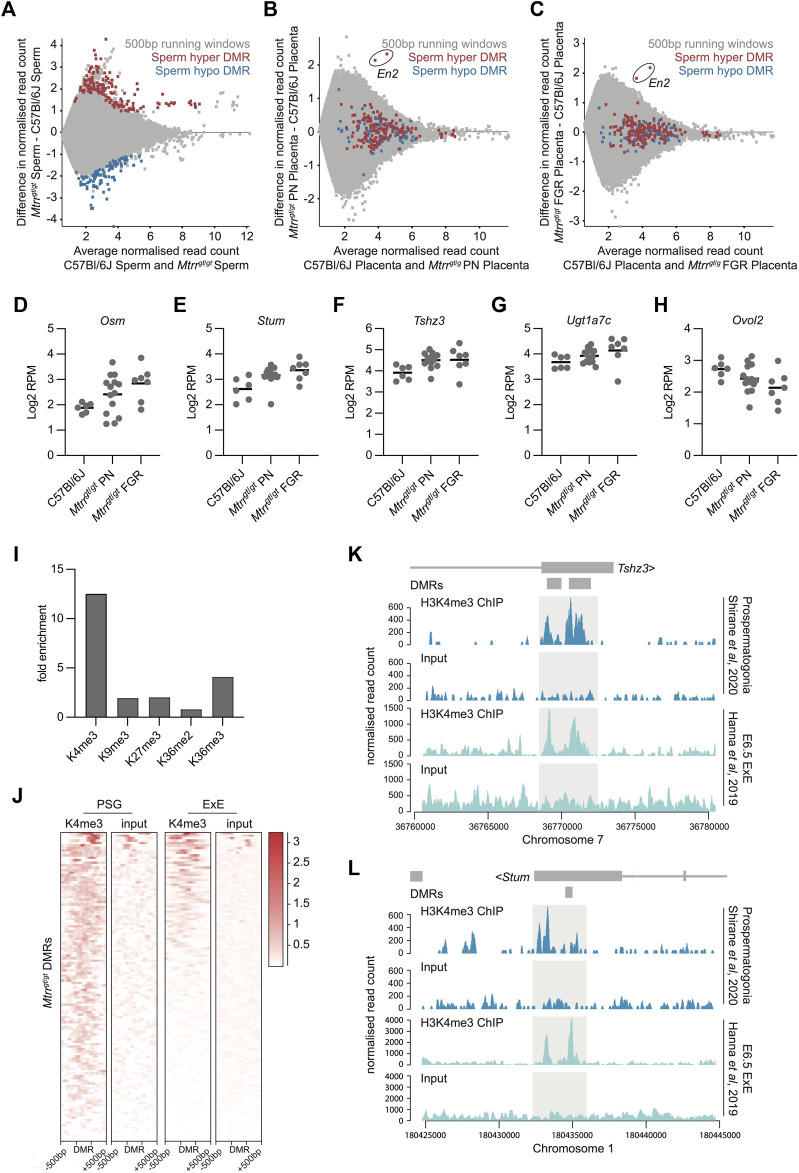
Spermatozoa DMRs in *Mtrr* ^*gt/gt*^ males were normalized in *Mtrr* ^*gt/gt*^ placentas yet associated with transcriptional dysregulation. **(A)** MA plot of *log*_*2*_ normalized meDIP-seq read counts of 500 bp contiguous regions in spermatozoa from C57Bl/6J and *Mtrr* ^*gt*/*gt*^ males. Hypermethylated (red) and hypomethylated (blue) differentially methylated 500 bp regions (DMRs) were identified using EdgeR. **(B)** MA plot of *log*_*2*_ normalized meDIP-seq read counts of 500 bp contiguous regions in placentas from C57Bl/6J and *Mtrr* ^*gt*/*gt*^ phenotypically normal fetuses at E10.5. The genomic regions where spermatozoa DMRs from *Mtrr* ^*gt/gt*^ males were identified are highlighted on the placenta data. Hypermethylated spermatozoa DMRs (red), hypomethylated spermatozoa DMRs (blue). The *En2* DMRs are indicated. **(C)** MA plot of *log*_*2*_ normalized meDIP-seq read counts of 500 bp contiguous regions in placentas from C57Bl/6J fetuses and *Mtrr* ^*gt*/*gt*^ fetal growth restricted (FGR) fetuses at E10.5. The genomic regions where spermatozoa DMRs from *Mtrr* ^*gt/gt*^ males were identified are highlighted on the placenta data. Hypermethylated spermatozoa DMRs (red), hypomethylated spermatozoa DMRs (blue). The *En2* DMRs are indicated. **(D–H)** Graphs showing placental transcript expression (*log*_*2*_RPM) of genes that were associated with spermatozoa DMRs including **(D)** *Osm*, **(E)** *Stum*, **(F)** *Tshz3*, **(G)** *Ugt1a7c*, and **(H)** *Ovol2*. Data was ascertained by RNA-seq of placentas from C57Bl/6J and *Mtrr* ^*gt*/*gt*^ conceptuses at E10.5. Placentas from phenotypically normal (PN) and fetal growth restricted (FGR) fetuses were assessed. **(I)** Enrichment for specific histone modifications in wildtype prospermatogonia ascertained by ChIP-seq at the 500 bp regions defined as DMRs in spermatozoa of *Mtrr* ^*gt*/*gt*^ males. Enrichment determined relative to the baseline genome. **(J)** Probe alignment plot showing H3K4me3 enrichment ascertained by ChIP-seq from wildtype prospermatogonia and extraembryonic ectoderm (ExE) at E6.5 compared to input controls in regions identified as spermatozoa DMRs (± 500 bp) in *Mtrr* ^*gt*/*gt*^ males. **(K,L)** Data tracks showing normalized H3K4me3 ChIP-seq reads and input controls for prospermatogonia (dark blue) and extraembryonic ectoderm (ExE) at E6.5 (light blue) in the regions surround the **(K)** *Tshz3* and **(L)** *Stum* sperm DMRs from *Mtrr* ^*gt/gt*^ males. Light grey boxes highlight H3K4me3 peaks. Small dark grey boxes indicate the DMRs. See also Supplementary File S3 for data sources. For spermatozoa meDIP-seq: C57Bl/6J, *N* = 8 males; *Mtrr* ^*gt/gt*^, *N* = 8 males. For placenta meDIP-seq: C57Bl/6J, *N* = 8 placentas; *Mtrr* ^*gt/gt*^ PN, *N* = 7 placentas; *Mtrr* ^*gt/gt*^ FGR, *N* = 7 placentas. For RNA-seq: C57Bl/6J, *N* = 6 placentas; *Mtrr* ^*gt/gt*^ PN, *N* = 14 placentas; *Mtrr* ^*gt/gt*^ FGR, *N* = 7 placentas.

In our previous study of epigenetic inheritance in the *Mtrr* ^*gt*^ mouse line (Blake et al., 2021), we found that a small number of candidate DMRs identified in mature spermatozoa (obtained from the cauda epididymis and vas deferens) were not recapitulated in embryos or placentas at E10.5 when interrogated by bisulfite pyrosequencing. Here, we aimed to validate this finding on a genome-wide scale by comparing our spermatozoa (Blake et al., 2021) and placenta meDIP-seq datasets from control and *Mtrr* ^*gt/gt*^ mice. First, we harmonised DMR calling between datasets by reanalysing the spermatozoa meDIP-seq datasets according to our analysis of the placenta meDIP-seq data. Hypermethylated spermatozoa DMRs in *Mtrr* ^*gt/gt*^ males that were previously identified in very highly methylated regions in control spermatozoa and described as false positives (Blake et al., 2021), were also clearly identifiable by the current analysis. Accordingly, we screened out these DMRs using whole genome bisulphite sequencing data to quantify absolute methylation levels across all DMRs (Sun et al., 2018). Similar to our candidate-based approach (Blake et al., 2021), DNA methylation patterns in nearly all genomic regions identified as spermatozoa DMRs in *Mtrr* ^*gt/gt*^ males were normal in *Mtrr* ^*gt/gt*^ placentas at E10.5 compared to control placentas (Figures 4A–C). This finding occurred regardless of the *Mtrr* ^*gt/gt*^ fetal growth phenotype. The only exception was the common hypermethylated *En2* DMR that appeared in both spermatozoa and placentas (Figures 4B, C). Conversely, the placental DMRs that overlap with RLTR4 elements were normally methylated in sperm of *Mtrr* ^*gt/gt*^ males (relative to control spermatozoa) suggesting that germline transposon silencing is maintained and unlikely to play a key role in epigenetic inheritance mechanisms in the *Mtrr* ^*gt*^ mouse line. Overall, these results indicated that placenta and spermatozoa DMRs in *Mtrr* ^*gt*/*gt*^ mice were tissue-specific and that most of the spermatozoa DMRs were effectively reprogrammed in the pre-implantation embryo or during placental development, notwithstanding the shared *Mtrr* ^*gt/gt*^ genotype of the parental and offspring generations. These data might negate DNA methylation as a mechanistic factor in epigenetic inheritance within the *Mtrr* ^*gt*^ mouse line. Instead, altered heritability of other epigenetic factors, such as histone modifications or small non-coding RNA content in germ cells, might be an alternative or additional mechanism.

Previously, our locus-specific analysis showed that some genes associated with spermatozoa DMRs were misexpressed in somatic tissues despite reprogramming of DNA methylation at these sites (Blake et al., 2021). Therefore, we questioned the extent to which this association occurred in the wider placental genome. Using DESeq, the *Mtrr* ^*gt/gt*^ placental RNA-seq dataset was assessed for differentially expressed genes that were within 2 kb of a sperm DMR from *Mtrr* ^*gt/gt*^ males and had transcript levels with a *log*_*2*_FC > 0.6 compared to control placentas. Five misexpressed genes (i.e., *Stum* (mechanosensory transducer mediator; membrane protein)*, Tshz3* (teashirt zinc finger family member 3; transcription factor)*, Ovol2* (ovo like zinc finger 2; transcription factor), *Osm* (oncostatin m; cytokine)*,* and *Ugt1a7c* (UDP glucouronosyltransferase 1 family, polypeptide A7C; enzyme in glucouronidation pathway) met these criteria but only in *Mtrr* ^*gt/gt*^ placentas with FGR (Figures 4D–H). The occurrence of transcriptional disruption despite normal DNA methylation reinforced our hypothesis that abnormal one-carbon metabolism influences other epigenetic mechanisms.

Next, we explored whether histone modifications were present in the developing germline at regions demarcated by spermatozoa DMRs to better understand the broader epigenetic context of these regions. To do this, ChIP-seq datasets were analysed for histone mark enrichment in developing wildtype male germ cells (i.e., prospermatogonia) (Shirane et al., 2020) at specific genomic regions defined by spermatozoa DMRs from *Mtrr* ^*gt/gt*^ males. First, we found that 103 out of 252 DMRs (40.9%) overlapped with an H3K4me3 peak in wildtype prospermatogonia, the majority which were located within gene bodies (Supplementary Table S1). This value represented a 12-fold enrichment compared to the baseline genome (i.e., only 3.3% of 500 bp regions across the whole genome overlapped with H3K4me3 peaks) and was substantially more enriched than the other histone modifications at the same locations (Figure 4I). Since H3K4me3 is typically associated with active transcription (Howe et al., 2017), it was an ideal candidate to further explore as an underlying inherited epigenetic mark associated with transcriptional disruption in the placenta. Therefore, we assessed whether wildtype trophoblast progenitor cells at E6.5 (i.e., extraembryonic ectoderm) displayed H3K4me3 enrichment at genomic locations identified as spermatozoa DMRs using a published ChIP-seq dataset (Hanna et al., 2019). Indeed, a substantial subset of these genomic regions was also enriched for H3K4me3 in extraembryonic ectoderm (Figure 4J). Remarkably, four out of five dysregulated genes in *Mtrr* ^*gt/gt*^ placentas that were associated with a spermatozoa DMR in *Mtrr* ^*gt/gt*^ males (i.e., *Stum*, *Tshz3*, *Ovol2*, *Ugta7c*) were among those enriched for H3K4me3 in both prospermatogonia and extraembryonic ectoderm (Figure 4K–L; Supplementary Figure S2). We infer from these data that the *Mtrr* ^*gt*^ allele potentially disrupts histone marks, such as H3K4me3, in developing and/or mature germ cells leading to altered patterns of the same histone mark in the early conceptus with implications for gene regulation. Indeed, we found that 59.0% of H3K4me3 peaks identified in extraembryonic ectoderm were also found in prospermatogonia (using MACS peak calling function embedded in SeqMonk software). This finding more broadly supports a role for H3K4me3 in inheritance of epimutations from germ cells to the placenta. Future mechanistic experiments should focus on multigenerational patterns of H3K4me3 in the *Mtrr* ^*gt*^ mouse line.

### 3.6 Mature male germ cell DMRs in *Mtrr* ^*+/gt*^ mice are not multigenerationally inherited

**FIGURE 5:**
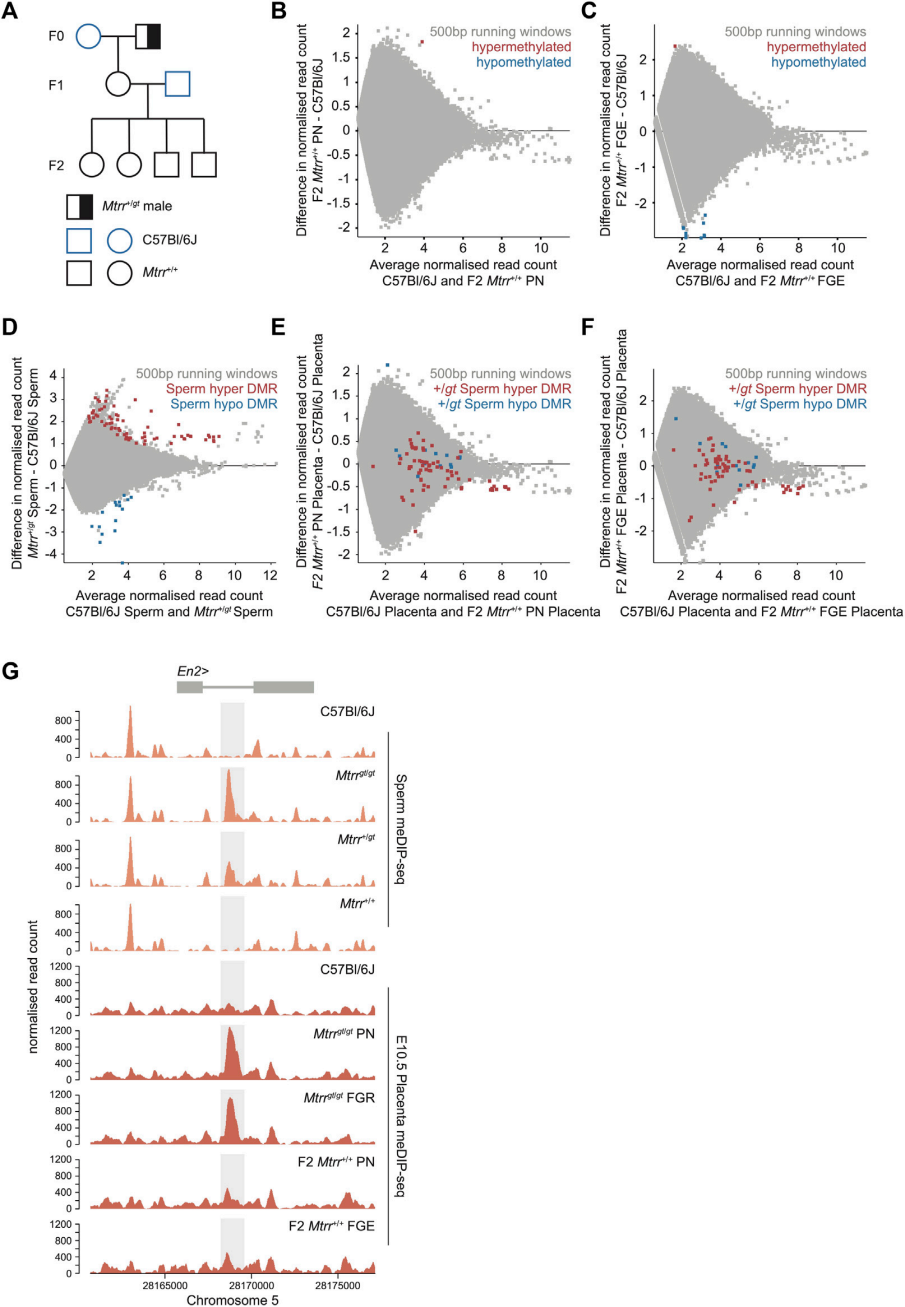
Spermatozoa DNA methylation patterns are not transgenerationally inherited in the *Mtrr* ^*gt*^ mouse line. **(A)** *Mtrr* ^+/gt^ maternal grandfather pedigree used in this study. Squares, males; circles females; blue outline, C57Bl/6J mouse line; black outline, *Mtrr* ^*gt*^ mouse line; white fill, *Mtrr* ^*+/+*^; half black/half white fill; *Mtrr* ^*+/gt*^. F0, parental generation; F1, first filial generation; F2, second filial generation. **(B)** MA plot of *log*_*2*_ normalized meDIP-seq read counts of 500 bp contiguous regions in placentas at E10.5 from C57Bl/6J conceptuses and F2 *Mtrr* ^+/+^ conceptuses derived from F0 *Mtrr* ^*+/gt*^ maternal grandfathers. Placentas from phenotypically normal (PN) fetuses were assessed. Hypermethylated (red) and hypomethylated (blue) differentially methylated regions (DMRs) were determined relative to control placentas using EdgeR. **(C)** MA plot of *log*_*2*_ normalized meDIP-seq read counts of 500 bp contiguous regions in placentas at E10.5 from C57Bl/6J conceptuses and F2 *Mtrr* ^+/+^ fetal growth enhanced (FGE) conceptuses derived from F0 *Mtrr* ^*+/gt*^ maternal grandfathers. Hypermethylated (red) and hypomethylated (blue) DMRs were determined using EdgeR. **(D)** MA plot of *log*_*2*_ normalized meDIP-seq read counts of 500 bp contiguous regions in spermatozoa from C57Bl/6J and F0 *Mtrr* ^*+/gt*^ males. Hypermethylated DMRs (red), hypomethylated DMRs (blue). **(E)** MA plot of *log*_*2*_ normalized meDIP-seq read counts of 500 bp contiguous regions in placentas from C57Bl/6J and F2 *Mtrr* ^*+/+*^ PN fetuses at E10.5. The genomic regions where spermatozoa DMRs from F0 *Mtrr* ^*+/gt*^ males were identified are highlighted on the placenta data. Hypermethylated spermatozoa DMRs (red), hypomethylated spermatozoa DMRs (blue). **(F)** MA plot of *log*_*2*_ normalized meDIP-seq read counts of 500 bp contiguous regions in placentas at E10.5 from C57Bl/6J fetuses and F2 *Mtrr* ^*+/+*^ FGE fetuses. The genomic regions where spermatozoa DMRs from F0 *Mtrr* ^*+/gt*^ males were identified are highlighted on the placenta data. Hypermethylated sperm spermatozoa m DMRs (red), hypomethylated spermatozoa DMRs (blue). **(G)** Data tracks across the *En2* gene showing normalized meDIP-seq read counts in spermatozoa (orange) from C57BL/6J males and *Mtrr* ^*gt*/*gt*^, *Mtrr* ^+/*gt*^ and *Mtrr* ^+/+^ males together with meDIP read counts in placentas at E10.5 (red) associated with C57Bl/6J fetuses, *Mtrr* ^*gt*/*gt*^ PN and FGR fetuses, and F2 *Mtrr* ^+/+^ PN and FGE fetuses. The *En2* DMR is highlighted in light grey. In all cases data was normalized to the largest data store. For spermatozoa meDIP-seq: C57Bl/6J, *N* = 8 males, F0 *Mtrr* ^*+/gt*^, *N* = 8 males. For placenta meDIP-seq: C57Bl/6J, *N* = 8 placentas; F2 *Mtrr* ^+/+^ PN, *N* = 8 placentas; F2 *Mtrr* ^+/+^ FGE, *N* = 3 placentas.

Our previous locus-specific analyses in F2 *Mtrr* ^*+/+*^ placentas indicated significant alteration of DNA methylation patterns caused by either a maternal grandfather or maternal grandmother *Mtrr* ^*gt*^ allele (Padmanabhan et al., 2013). Here, a transgenerational mechanism was explored in the *Mtrr* ^*+gt*^ maternal grandfather pedigree (Figure 5A) using a genome-wide approach to identify the locations of spermatozoa DMRs from F0 *Mtrr* ^*+/gt*^ males (Blake et al., 2021) and determine whether the placental methylome and transcriptome was altered in these regions two generations later in the F2 *Mtrr* ^*+/+*^ grandprogeny. The following matings were performed to generate this pedigree (Figure 5A): F0 *Mtrr* ^*+/gt*^ males were mated with C57Bl/6J control females, and the resulting F1 *Mtrr* ^*+/+*^ females were selected for mating with C57Bl/6J males to generate F2 *Mtrr* ^*+/+*^ conceptuses. F2 *Mtrr* ^*+/+*^ conceptuses were rigorously phenotyped at E10.5, and the placentas from PN and FGE fetuses were examined. First, the broader methylome of whole F2 *Mtrr* ^*+/+*^ placentas was assessed via meDIP-seq. When compared with C57Bl/6J controls, there were no significant differences in the distribution of meDIP reads across genomic features (Supplementary Figure S1C) and no clustering of biological replicates according to phenotype or pedigree (Supplementary Figure S1D) indicating similar global methylation among experimental groups. Few DMRs were identified by the meDIP-seq analysis including one hypermethylated DMR in F2 *Mtrr* ^*+/+*^ placentas from PN fetuses (Figure 5B) and 11 DMRs (10 hypomethylated, 1 hypermethylated) in FGE-associated F2 *Mtrr* ^*+/+*^ placentas (Figure 5C). Closer analysis revealed that 9 out of 10 of the hypomethylated DMRs from F2 *Mtrr* ^*+/+*^ FGE placentas were clustered in two locations on chromosomes 14 and 17, which are frequently susceptible to mapping artefacts in our datasets and so were excluded. The remaining three placental DMRs from F2 *Mtrr* ^*+/+*^ placentas were in nondescript genomic regions (Supplementary File S1). Importantly, the RLTR4 elements identified in *Mtrr* ^*gt/gt*^ placentas (Figure 3) exhibited normal levels of DNA methylation and transcript expression in the F2 *Mtrr* ^+/+^ placentas relative to control placentas (Supplementary Figure S3). This result suggested that the changes in DNA methylation described in *Mtrr* ^*gt*/*gt*^ placentas are intrinsically associated with the *Mtrr* ^*gt*^ allele and are unlikely to be transgenerationally inherited or caused by genetic differences between the C57Bl/6J control and *Mtrr* ^*gt*^ mouse lines.

When the meDIP-seq datasets from spermatozoa of F0 *Mtrr* ^*+/gt*^ males (Figure 5D) (Blake et al., 2021) were compared to placentas of F2 *Mtrr* ^*+/+*^ conceptuses (PN and FGE) at E10.5, there was no DMR overlap (Figures 5E, F). This finding reinforces our hypothesis that specific DMRs in the *Mtrr* ^*gt*^ mouse line are not inherited from germline to somatic cells over multiple generations. This was even the case at the *En2* DMR, which was present in spermatozoa from F0 *Mtrr* ^*+*/*gt*^ males and not in F2 *Mtrr* ^*+/+*^ placentas (Figure 5G). In this context, the spermatozoa and placenta methylome data revealed that the dosage of the *Mtrr* ^*gt*^ allele in mice correlated with the degree of hypermethylation at the *En2* DMR (Figure 5G). Therefore, the *En2* locus was particularly responsive to *Mtrr*-driven disruption of one-carbon metabolism. Altogether, these data further separate the transmission of specific differential methylation patterns via the germline from fetal growth phenotype inheritance in the *Mtrr* ^*gt*^ mouse line.

## 4 Discussion

Despite its well-studied role in development and disease, the molecular function of one-carbon metabolism is complex and not well understood. Here, we used the *Mtrr* ^*gt*^ mouse line to explore the epigenetic role of one-carbon metabolism by assessing the placental methylome in association with fetal growth phenotypes. In doing so, we identified several genomic regions in *Mtrr* ^*gt/gt*^ placentas with altered DNA methylation including in a gene promoter that conceivably regulates *Cxcl1* gene expression in a canonical manner, in a presumptive developmental regulatory region located within the *En2* gene, and in a subset of RLTR4 transposable elements. While unlikely to underlie the fetal growth phenotypes, it is possible that these DNA methylation changes are functionally relevant in other tissue types and/or for driving other phenotypes. For instance, CXC chemokine expression from peripheral blood mononuclear cells correlates with folate and homocysteine levels in human subjects (Holven et al., 2002). Alternatively, knocking out the mouse gene *Mtfhr* to disrupt folate metabolism causes cerebellar patterning defects that are associated with downregulation of *En2* gene expression (Chen et al., 2005). Ultimately, our findings support widespread epigenetic instability in the *Mtrr* ^*gt*^ mouse line.

Our previous locus-specific analyses indicated that *Mtrr* ^*gt/gt*^ placentas or wildtype placentas exposed to a maternal grandparental *Mtrr* ^*gt*^ allele are epigenetically unstable (Padmanabhan et al., 2013; Bertozzi et al., 2021; Blake et al., 2021). Yet, we identified fewer placenta DMRs by meDIP-seq than were expected despite using standard analysis parameters that yielded many spermatozoa DMRs in *Mtrr* ^*gt/gt*^ mice (e.g., 13 placenta DMRs vs. 252 spermatozoa DMRs). It is possible that placental DNA methylation is less sensitive than male germ cells to impaired one-carbon metabolism. DNA in trophoblast cells is globally hypomethylated compared to other cell types (Senner et al., 2012) and changes in DNA methylation might be less striking in this context. The use of whole placentas that contain multiple cell types (e.g., trophoblast cell subtypes, fetal vascular endothelium, and maternal decidua and immune cells) with their own DNA methylation and transcriptional signatures (Vento-Tormo et al., 2018; Andrews et al., 2023) might confound our analysis to some extent. The placental DMRs that we identified are likely present throughout the tissue, while other undetected DMRs may be confined to a single cell type and not appreciated in our analysis. Assaying the placenta at earlier developmental time points when the trophoblast progenitor population is more homogeneous may be informative. Alternatively, single cell-based sequencing methods may uncover additional DMRs in *Mtrr* ^*gt/gt*^ placentas at E10.5 that correlate with cell-type specific transcriptional dysregulation and phenotypes.

Transposable elements, which make up ∼40% of the mammalian genome (Goodier and Kazazian, 2008), are heavily methylated to suppress transposition causing deleterious mutation. In this study, we observed hypomethylation and ectopic expression of several RLTR4 elements in *Mtrr* ^*gt/gt*^ placentas, which might have profound consequences to genomic stability during development. While whole genome sequencing revealed that *de novo* mutation rates are similar in control and *Mtrr* ^*gt/gt*^ mice (Blake et al., 2021), it is still possible that increased transposition might occur in this context, generating structural variation with implications for phenotype inheritance. Since spermatozoa from *Mtrr* ^*gt/gt*^ males showed normal RLTR4 DNA methylation, we propose that DNA methylation was poorly maintained in early embryogenesis or in placenta development to cause hypomethylation at these sites. Although RLTR4 elements were the only transposons identified by meDIP-seq in this study, DNA methylation patterns of variably methylated intracisternal A particle (VM-IAP) retrotransposons are also considerably shifted in *Mtrr* ^*gt/gt*^ mice as determined by bisulfite pyrosequencing (Bertozzi et al., 2021). KRAB-ZPFs are known to regulate VM-IAPs (Bertozzi et al., 2020), and mechanistically, the *Mtrr* ^*gt*^ locus contains a cluster of 129P2Ola/Hsd-derived KRAB zinc finger proteins (ZFPs) in an otherwise C57Bl/6J background because of the mutagenesis process (Bertozzi et al., 2021). However, the KRAB-ZFP clusters within the *Mtrr* locus do not appear to regulate RLTR4 expression (Wolf et al., 2020). Others have shown that paternal *Mthfr* deficiency in mice causes hypomethylation of L1Md subfamily of LINE-1 retrotransposons (Karahan et al., 2021). Altogether, these data highlight the importance of one-carbon metabolism in maintaining epigenetic stability at early developmental stages when deleterious transposition events could have profound consequences.

While the mechanistic understanding of epigenetic inheritance remains in its infancy, several candidate epigenetic factors have been identified (e.g., chromatin modifications, small non-coding RNA content in germ cells) (Blake and Watson, 2016; Hanna et al., 2019). Our data provides genome-wide evidence that nearly all spermatozoa DMRs caused by the *Mtrr* ^*gt*^ allele were epigenetically reprogrammed in the placenta and were not transgenerationally inherited. This contrasts with another study that demonstrates transgenerational inheritance of directed epimutations of DNA methylation in mouse obesity genes along with an obesity phenotype, despite evidence that these epimutations are reprogrammed in primordial germ cells (Takahashi et al., 2023). The lack of DMR inheritance in the *Mtrr* ^*gt*^ mouse line suggests that there might be paradigm-specific effects. However, there are clues that spermatozoa DMRs caused by an *Mtrr* ^*gt*^ allele might still play a role in epigenetic inheritance since they are associated locus-specific disruption of transcription in *Mtrr* ^*gt/gt*^ placentas (this study) and in F2 *Mtrr* ^*+/+*^ embryos and adult livers (Blake et al., 2021) despite being reprogrammed to normal tissue-specific methylation levels. This association evokes a role for other epigenetic mechanisms aside from DNA methylation in epigenetic inheritance mechanisms. We observed enrichment for the activating H3K4me3 histone mark in developing wildtype male germ cells and trophoblast specifically at genomic locations defined by spermatozoa DMRs in *Mtrr* ^*gt/gt*^ mice including loci associated with *Mtrr* ^*gt/gt*^ placental gene misexpression. Others have shown a similar association in a mouse model of paternal *Mthfr* deficiency (Karahan et al., 2021).

We propose a model whereby impaired one-carbon metabolism alters H3K4me3 deposition in developing male germ cells, which then drives the changes in DNA methylation through modified access of DNA methyltransferases. In the pre-implantation embryo when DNA methylation is reprogrammed, a subset of abnormal H3K4me3 marks may persist, driving further changes in establishing aberrant *de novo* DNA methylation patterns or in gene expression in early cell lineages of the placenta and/or embryo. The most drastic epigenetic changes likely lead to altered lineage decisions and developmental phenotypes. Since a wide spectrum of phenotypes are observed in *Mtrr* ^*gt*^ mouse line, disrupting one-carbon metabolism might cause stochastic epigenetic changes across the genome, affecting different cell types in different individuals. The most striking data that reinforces a potential role for histone H3K4me in epigenetic inheritance comes from a study whereby wildtype mice were fed a folate-deficient diet. Mature spermatozoa from folate-deficient males displayed alterations in histone H3K4me3 patterns specifically at developmental genes and putative enhancers, a subset of which were retained in the F1 8-cell embryos and were associated with gene misexpression (Lismer et al., 2020). We did not observe any discernable changes in H3K4me3 enrichment in spermatozoa or 8-cell embryos derived from folate-deficient males specifically within the genomic regions identified as spermatozoa DMRs from *Mtrr* ^*gt*/*gt*^ males. This may be due to the differences in the mouse models employed, with our genetic approach causing a more severe metabolic effect than dietary deficiency. Regardless, this finding suggests that genomic hotspots regulated by one-carbon metabolism are unlikely and that the epigenome is differently or stochastically affected in these models. To fully understand the mechanisms involved in epigenetic inheritance, histone methylation should be explored as an inherited epigenetic mechanism in the *Mtrr* ^*gt*^ mouse model.

Overall, this study together with our previously published work (Padmanabhan et al., 2013; Bertozzi et al., 2021; Blake et al., 2021) indicate that one-carbon metabolism is required for the maintenance of epigenetic stability in the placenta and the germline. The widespread effect of disrupting one-carbon metabolism on the epigenome provides some explanation towards the complex molecular role of folate metabolism during development. Instability of the epigenome can alter transcriptional pathways and genomic stability, with substantial downstream effects on developmental outcome. Single-cell sequencing technology and a broader analysis of epigenetic mechanisms (e.g., histone marks and chromatin structure) together with DNA methylation will enable the identification of complex epigenome-phenotype relationships that persist over multiple generations in context of the *Mtrr* ^*gt*^ mouse line.

## Data Availability

The original data presented in the study are deposited in the GEO repository, accession number GSE233482. The published datasets presented in this study can be found in online repositories. The names of the repository and the accession numbers can be found in Supplementary File S3.
