# Supplementary material for: One-carbon metabolism is required for epigenetic stability in the mouse placenta
Source: Front Cell Dev Biol. 2023 Jun 27;11:1209928. doi: 10.3389/fcell.2023.1209928 (PMC10333575; doi:10.3389/fcell.2023.1209928)
Supplement: Supplementary file 3 [file Table1.DOCX]

Supplementary Material

One-carbon metabolism is required for epigenetic stability in the mouse placenta

Claire E. Senner^*^, Ziqi Dong, Malwina Prater, Miguel R. Branco, Erica D. Watson^*^

*** Correspondence:** Erica Watson: [edw23@cam.ac.uk](mailto:edw23@cam.ac.uk); Claire Senner: [ces207@cam.ac.uk](mailto:ces207@cam.ac.uk)

# Supplementary Data

**Supplementary Data File 1:** Genomic regions of hypomethylated (hypo) and hypermethylated (hyper) differentially methylated regions identified via meDIP-seq in placentas at E10.5 of *Mtrr^gt/gt^* conceptuses (phenotypically normal [PN] and fetal growth restricted [FGR]) and of F2 generation *Mtrr^+/+^* conceptuses (PN and fetal growth enhanced [FGE]) derived from F0 generation *Mtrr^+/gt^* males and F1 generation *Mtrr^+/+^* females.

**Supplementary Data File 2:** *RLTR4_Mm* and *RLTR4_Mm-int* genomic regions identified via meDIP-seq in placentas at E10.5 from *Mtrr^gt/gt^* conceptuses.

**Supplementary Data File 3:** Accession numbers and citations of processed data analyzed in this study.

# Supplementary Tables

**Supplementary Table 1.** Genomic distribution of all mature spermatozoa DMRs in *Mtrr^gt/gt^* males and those that overlapped with H3K4me3 peaks in wildtype prospermatogonia.

|  | Promoters  (-2 kb to +200 bp relative to TSS) | Gene body | Intergenic |
| --- | --- | --- | --- |
| Spermatozoa DMRs from *Mtrr^gt/gt^* males (252 DMRs total) | 7.5% (19/252) | 38.9% (98/252) | 53.6% (135/252) |
| Spermatozoa DMRs from *Mtrr^gt/gt^* males that overlap with H3K4me3 peaks in wildtype prospermatogonia (103 DMRs total) | 15.5% (16/103) | 62.1% (64/103) | 22.3% (23/103) |

DMRs, differentially methylated regions; H3K4me3, histone 3 lysine 4 trimethylation; TSS, transcriptional start site

# Supplementary Figures

**Supplementary Figure 1.** Distribution of meDIP-seq reads across genomic features and phenotypes.

(**A)** Enrichment of meDIP-seq reads (*log_2_* scale) across genomic features relative to IgG in C57Bl/6J placentas, and *Mtrr^gt^*^/^*^gt^* placentas from conceptuses that were phenotypically normal (PN) or displayed fetal growth restriction (FGR) at E10.5. **(B)** Data Store tree displaying all replicates from C57Bl/6J, *Mtrr^gt^*^/^*^gt^* PN, and *Mtrr^gt^*^/^*^gt^* FGR placentas. Clustering is based on normalised read counts of 500 bp contiguous regions tiled over the genome. **(C)** Enrichment of meDIP-seq reads (*log_2_* scale) across genomic features relative to IgG in C57Bl/6J placentas at E10.5 and F2 *Mtrr*^+/+^ placentas at E10.5 from PN or fetal growth enhanced (FGE) conceptuses derived from an F0 *Mtrr^+/gt^* maternal grandfather. **(D)** Data Store tree displaying all replicates from C57Bl/6J placentas and F2 *Mtrr*^+/+^ placentas from PN and FGE conceptuses. Clustering is based on normalised read counts of 500 bp contiguous regions tiled over the genome. CGIs, CpG islands.

**
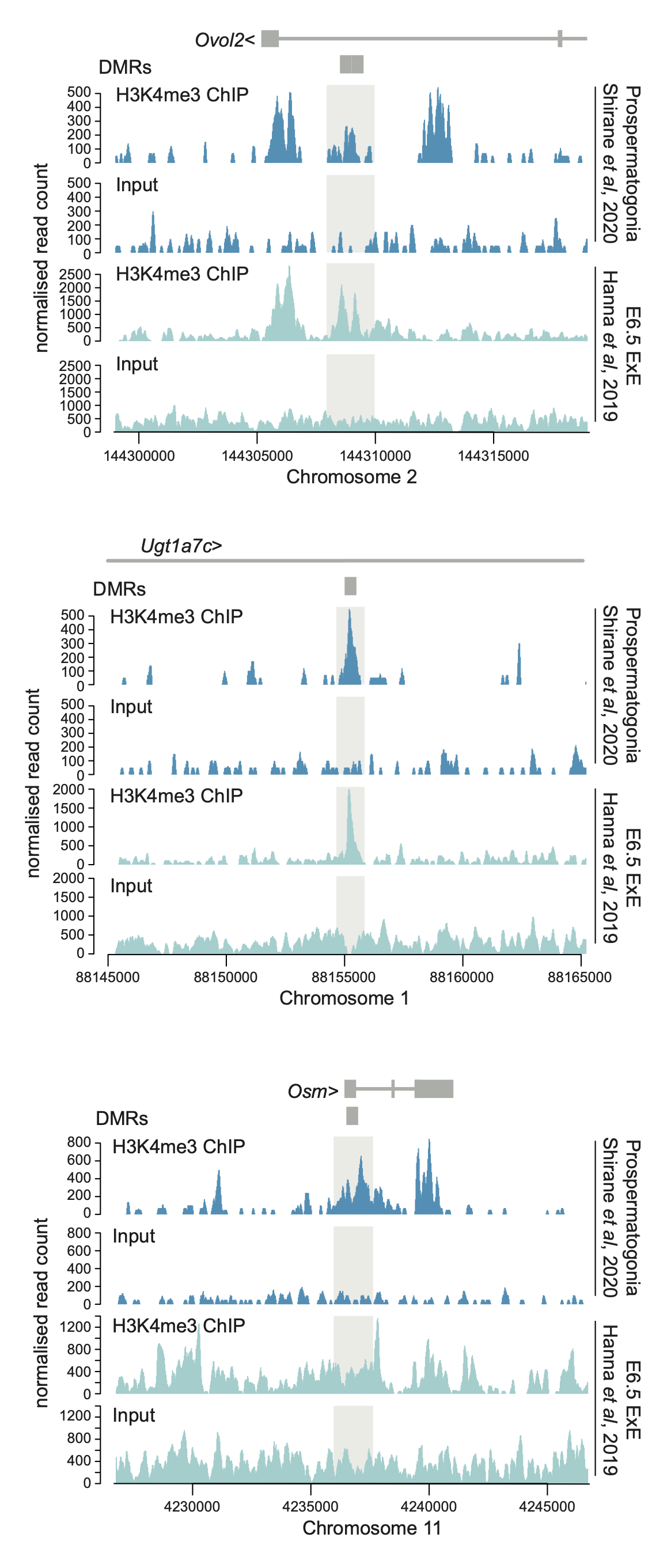
**

**Supplementary Figure 2.** H3K4me3 enrichment in prospermatogonia and extraembryonic ectoderm in regions defined by spermatozoa DMRs and placental gene misexpression in *Mtrr^gt/gt^* mice.

Data tracks showing H3K4me3 ChIP-seq reads and input controls for prospermatogonia (dark blue peaks) and extraembryonic ectoderm (ExE) at E6.5 (light blue peaks). The regions shown are associated with mature spermatozoa DMRs (dark grey) identified in *Mtrr^gt/gt^* males that correspond to gene misexpression in *Mtrr^gt/gt^* placentas at E10.5 including (**top panel**) *Ovol2* DMR, (**middle panel**) *Ugt1a7* DMR, and (**bottom panel**) *Osm* DMR. Light grey boxes highlight H3K4me3 peaks within the region specified as a spermatozoa DMR from *Mtrr^gt/gt^* mice. See also Supplementary Data File 3 for data sources.


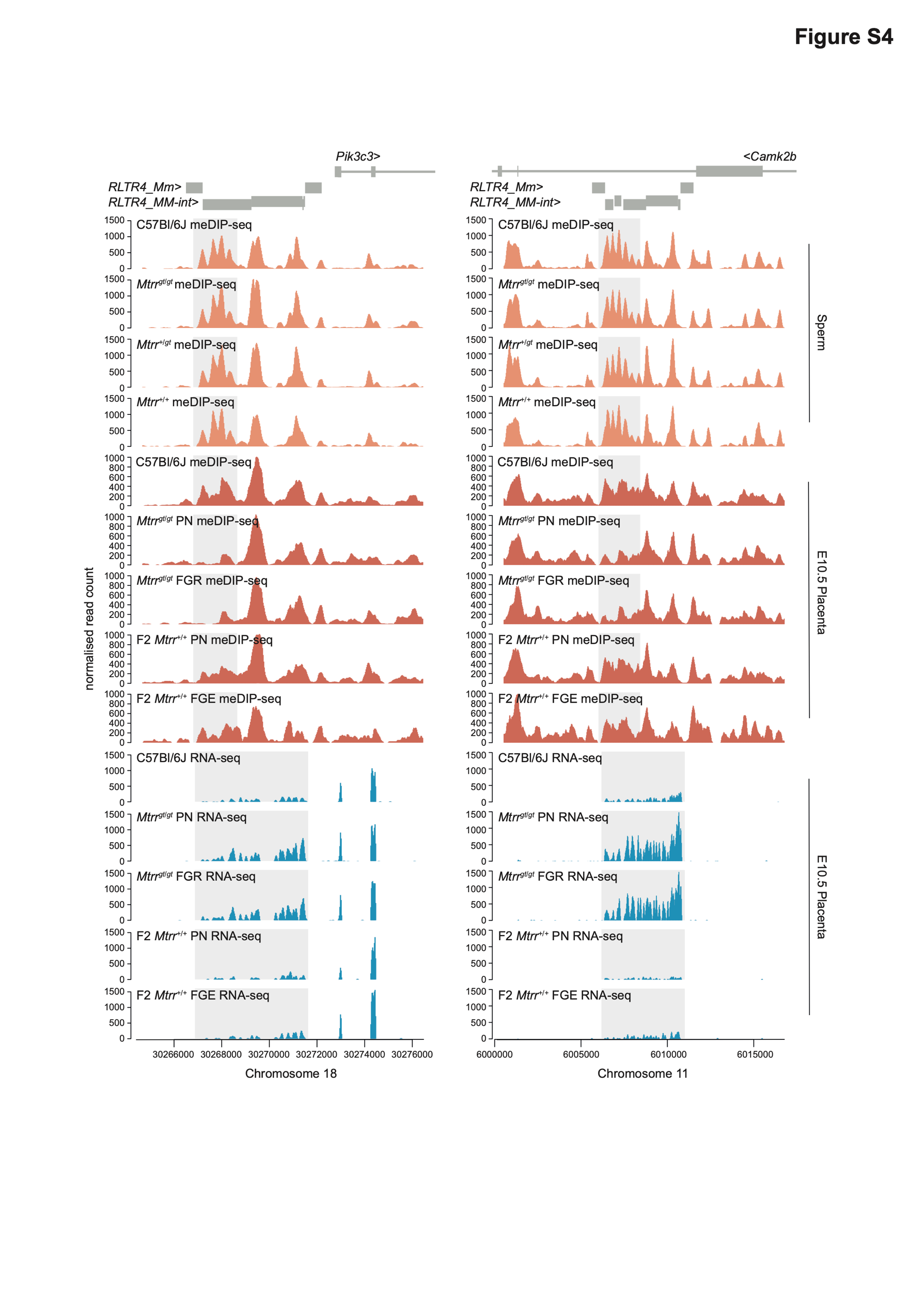


**Supplementary Figure 3.** DNA methylation and transcript levels at specific RLTR4 elements in spermatozoa and placenta at E10.5 from the *Mtrr^gt^* mouse line.

Data tracks showing normalised meDIP-seq and RNA-seq reads across full-length ERVs comprising *RLTR4_Mm* and *RLTR4_MM-int* elements on (**left-hand panel**) mouse chromosome 18 associated with the *Pik3c3* gene and (**right-hand panel**) chromosome 11 associated with the *Camk2b* gene. Spermatozoa meDIP-seq data was from adult sperm of C57Bl/6J, *Mtrr^gt/gt^*, *Mtrr^+/gt^*, and *Mtrr^+/+^* mice (orange). Placenta meDIP-seq (red) and RNA-seq (blue) data was from placentas at E10.5 of C57Bl/6J conceptuses, *Mtrr^gt^*^/^*^gt^* conceptuses associated with phenotypically normal (PN) or fetal growth restricted (FGR) fetuses, and F2 *Mtrr^+/+^* conceptuses associated with PN or fetal growth enhanced (FGE) fetuses. Genomic locations of the DMRs and differential transcript expression is highlighted in light grey. N values: spermatozoa meDIP-seq: N=8 per experimental group; placenta meDIP-seq: C57Bl/6J, N=8 placentas; *Mtrr^gt/gt^* PN, N=7 placentas; *Mtrr^gt/gt^* FGR, N=7 placentas; F2 *Mtrr^+/+^* PN, N=8 placentas; F2 *Mtrr^+/+^* FGE, N=3 placentas; placenta RNA-seq: C57Bl/6J, N=6 placentas; *Mtrr^gt/gt^* PN, N=14 placentas; *Mtrr^gt/gt^* FGR, N=7 placentas; F2 *Mtrr^+/+^* PN, N=4 placentas; F2 *Mtrr^+/+^* FGE, N=4 placentas.
